# Pleiotropy of Alzheimer’s Disease and Educational Attainment: Insights from the Summary Statistics

**DOI:** 10.1093/geroni/igab046.3513

**Published:** 2021-12-17

**Authors:** Yury Loika, Elena Loiko, Irina Culminskaya, Alexander Kulminski

**Affiliations:** 1 Duke University, Durham, North Carolina, United States; 2 DUKE UNIVERSITY, Durham, North Carolina, United States

## Abstract

Epidemiological studies report beneficial associations of higher educational attainment (EDU) with Alzheimer’s disease (AD). Prior genome-wide association studies (GWAS) also reported variants associated with AD and EDU separately. The analysis of pleiotropic predisposition to these phenotypes may shed light on EDU-related protection against AD. We examined pleiotropic predisposition to AD and EDU using Fisher’s method and omnibus test applied to summary statistics for single nucleotide polymorphisms (SNPs) associated with AD and EDU in large-scale univariate GWAS at suggestive-effect (5×10-8

